# Neuroprosthetic-enabled control of graded arm muscle contraction in a paralyzed human

**DOI:** 10.1038/s41598-017-08120-9

**Published:** 2017-08-21

**Authors:** David A. Friedenberg, Michael A. Schwemmer, Andrew J. Landgraf, Nicholas V. Annetta, Marcia A. Bockbrader, Chad E. Bouton, Mingming Zhang, Ali R. Rezai, W. Jerry Mysiw, Herbert S. Bresler, Gaurav Sharma

**Affiliations:** 1 Advanced Analytics and Health Research, Battelle Memorial Institute, 505 King Avenue, Columbus, Ohio, 43201 USA; 2 Medical Devices and Neuromodulation, Battelle Memorial Institute, 505 King Avenue, Columbus, Ohio, 43201 USA; 3 Center for Neuromodulation, The Ohio State University, Columbus, Ohio, 43210 USA; 4 Department of Physical Medicine and Rehabilitation, The Ohio State University, Columbus, Ohio, 43210 USA; 5 Department of Neurological Surgery, The Ohio State University, Columbus, Ohio, 43210 USA; 6 Present Address: Feinstein Institute for Medical Research, 350 Community Drive, Manhasset, New York, 11030 USA

## Abstract

Neuroprosthetics that combine a brain computer interface (BCI) with functional electrical stimulation (FES) can restore voluntary control of a patients’ own paralyzed limbs. To date, human studies have demonstrated an “all-or-none” type of control for a fixed number of pre-determined states, like hand-open and hand-closed. To be practical for everyday use, a BCI-FES system should enable smooth control of limb movements through a continuum of states and generate situationally appropriate, graded muscle contractions. Crucially, this functionality will allow users of BCI-FES neuroprosthetics to manipulate objects of different sizes and weights without dropping or crushing them. In this study, we present the first evidence that using a BCI-FES system, a human with tetraplegia can regain volitional, graded control of muscle contraction in his paralyzed limb. In addition, we show the critical ability of the system to generalize beyond training states and accurately generate wrist flexion states that are intermediate to training levels. These innovations provide the groundwork for enabling enhanced and more natural fine motor control of paralyzed limbs by BCI-FES neuroprosthetics.

## Introduction

Spinal cord injury (SCI) is one of the leading causes of paralysis worldwide. However, in most SCI cases, even though the signal pathways between the brain and the limbs might be disrupted, both the motor cortex and the limb muscles that it controls remain intact. Research in the field of brain computer interfaces (BCIs) has consistently demonstrated that brain activity in the intact motor cortex can be leveraged to establish a direct, functional connection to assistive devices that can restore motor and sensory functions^1–4^. More recent work has shown that signals recorded from the brain can be used to directly activate paralyzed muscles via functional electrical stimulation (FES)^5–11^. In humans, BCI control of FES (BCI-FES) has enabled control of hand movements in paralyzed participants^8, 9^. However, these studies have only demonstrated control of a fixed number of pre-determined discrete movements. Graded muscle contraction, which is necessary for performing fine motor tasks such as grasping an egg without breaking it, has not yet been demonstrated in paralyzed humans. Thus, the ability to voluntarily grade muscle contraction using a BCI-FES system is a necessary step toward allowing SCI patients to regain more natural control of hand usage, which patients report as the most desirable function to regain^12^.

Intracortical recordings from primates have provided ample evidence that neurons in the primary motor cortex (M1) carry information related to graded muscle activation and force exertion^13–15^, and these signals have even been used to predict electromyography activity (EMG) and control electrical stimulation of primate arm muscles^5–7, 11, 16^. For example, Moritz *et al*.^7^ trained monkeys to modulate the activity of 1-2 neurons to achieve limited force generation in 1 or 2 wrist muscles, while Pohlmeyer *et al*.^6^ showed that, using a cortically controlled FES system, monkeys can control the contraction of forearm muscles and use it to match a cursor to targets at different discrete force levels. Similar to primate studies, functional imaging experiments in humans have shown that increasing force production is associated with linear increases in blood oxygen level-dependent (BOLD) signals in contralateral M1 and medial motor regions^17–19^. More recently, Murphy *et al*.^20^ demonstrated that stereoelectroencephalographic depth electrodes implanted in deep brain structures could discriminate between rest and force states in epileptic patients, and they also attempted to discriminate between “rest,” “light,” and “hard” force levels, but were only significantly above chance classification for discriminating between the light and hard force levels 25% of the time. Similarly, Shah *et al*.^21^ demonstrated that force onset, holding, and release states can be extracted from local field potentials recorded with deep brain electrodes in Parkinson’s patients, but their work did not attempt to decode the magnitude of the force. Although several FES systems have been shown to restore discrete grasping motions in paralyzed individuals^8–10^, it has not yet been demonstrated that muscle activity can be reliably extracted from M1 neurons and used to control the graded activation of limb muscles.

In this study, we demonstrate two important advances toward restoring graded muscle contraction in paralyzed humans. First, we show that information related to graded muscle contraction intent is encoded in the motor cortex and can be accurately decoded from intracortically recorded data. Second, we report that a tetraplegic human can use this information and a BCI-controlled, non-invasive FES to regain volitional control of graded muscle contraction in his paralyzed limb. We observed distinct neural activity as the participant was asked to imagine exerting force to control an animated needle on a computer screen and match it to graded cue angles on a virtual protractor. The participant was then able to control the amplitude of FES to his own muscles to generate graded wrist flexion force against a resistance using his own thoughts, including generalizing to positions not attempted during training. In addition to providing insights into the relationship between cortical neuronal activity and muscle contraction during voluntary hand movements, our results also demonstrate an important advancement of neuroprosthetic technology that can help people suffering from paralysis regain the voluntary and smooth graded control of muscle activation that is necessary for dexterous manipulation of delicate objects.

## Methods

### Study Participant

The study participant was a 27-year-old male with stable, non-spastic C5/C6 quadriplegia from cervical SCI. He underwent implantation of a Utah microelectrode array (Blackrock Microsystems, Inc., Salt Lake, Utah) in his left primary motor cortex. The hand area of motor cortex was identified preoperatively by fusing functional magnetic resonance imaging (fMRI) activation maps obtained while the patient attempted movements co-registered to the preoperative planning MRI. Full details of the fMRI and surgical procedures can be found in Bouton *et al*.^9^. Approval for this study was obtained from the U.S. Food and Drug Administration (Investigational Device Exemption) and the Ohio State University Medical Center Institutional Review Board (Columbus, Ohio). The study met institutional requirements for the conduct of human subjects and was registered on the ClinicalTrials.gov website (Identifier NCT01997125; Date: November 22, 2013). All experiments were performed in accordance with the relevant guidelines and regulations set by the Ohio State University Medical Center. The participant referenced in this work provided permission for photographs and videos and completed an informed consent process prior to commencement of the study.

### System Architecture

The Neuroport neural data acquisition system and the Utah microelectrode array with electrodes 1.5 mm long (Blackrock Microsystems, Inc., Salt Lake City, Utah) were used to acquire the neural data. A 0.3 Hz first order high-pass and a 7.5 kHz third order low-pass Butterworth analog hardware filter was applied to the data, and each of the 96 channels of the microelectrode array were sampled at 30 kHz. The digitized data were then transmitted to a PC where they were decoded to determine how much force to exert and then encoded to evoke the desired response from the muscles in the forearm. Simultaneously, the PC communicated with the custom high-definition neuromuscular electrical stimulator that drove the electrode sleeve wrapped around the forearm.

### Signal Processing and Decoding

The signal processing and decoding/control algorithms were all run on a PC using MATLAB 2012a. The digitized data from the Neuroport was processed every 100 ms. Stimulation artifacts in the data were detected using by threshold crossings of 500 µV occurring simultaneously on at least 4 of 12 randomly selected channels. A 3.5 ms window of data around each detected artifact was then removed and adjacent data segments were rejoined. This approach removes most of the artifact while leaving a minimum of 82.5% of the original data, identical to the work of Bouton *et al*.^9^ but with a larger window.

Neural activity was measured using mean wavelet power (MWP), also similar to Bouton *et al*.^9^. Wavelet decomposition was applied to the raw voltage data, using the ‘db4’ mother wavelet and 11 wavelet scales. Four wavelet scales^3–6^ were used, corresponding to the multiunit frequency band spanning approximately 234 to 1875 Hz. The mean of the wavelet coefficients for each scale of each channel was calculated every 100 ms and a 1 s wide boxcar filter was applied to smooth the data. Baseline drift in the data was estimated by using a 15 s boxcar filter and was subtracted from the smoothed mean wavelet coefficients for the corresponding 100 ms window (this subtraction was only applied during the imagined graded control of muscle contraction experiment). The mean coefficients were then standardized per channel, per scale, by subtracting the mean and dividing by the standard deviation of those scales and channels during the training blocks. The four scales were then combined by averaging the standardized coefficients for each channel, resulting in 96 MWP values, one for each electrode in the array, for every 100ms of data. The resulting MWP values were used as features and input into the decoder.

Two different decoding algorithms were used to map neural modulation, as measured by MWP, to the angle the user intended to point toward. First, a beta regression^22^ allowed for straightforward evaluation of the contribution of each channel to the model using standard hypothesis tests. Angles from 0 to 180 degrees were scaled to the unit interval and then fit to the training data using a logit link function so that the logit of the scaled angle $\mu$ is modeled as a linear combination of the 96 MWP features plus an intercept term.

$$logit(\mu )={\beta }_{0}+\,\sum _{j=1}^{96}\,{\beta }_{j}MW{P}_{j}$$

Maximum likelihood estimates of the parameters as well as hypothesis tests are calculated assuming a beta distribution of the response variable. The resulting model is similar to a logistic regression model in that the logit of the mean is modeled as a linear function of the covariates, but beta regression maximizes a different likelihood, owing to the fact that the data lie on the continuous range between 0 and 1, as opposed to binary for a logistic model. The model can then predict the intended scaled angle from a new set of 96 MWP values which can then be displayed on screen or translated to a stimulation pattern. Models were fitted using a modified version of the beta regression package^23^ using MATLAB 2012a.

The second decoding algorithm used is the *libsvm* implementation^24^ of the Support Vector Regression (SVR) algorithm^25^. SVR is the regression counterpart of the popular Support Vector Machine classification algorithm that has been used successfully in previous BCI studies^9, 26, 27^. SVR has\ two main advantages over the beta regression and one disadvantage. First, SVR is better suited to handle non-linear relationships and interactions between the channels because the data are transformed to a high-dimensional space using a non-linear kernel. Second, the SVR algorithm has a built-in regularization parameter that minimizes the impact of channels that do not contribute useful information to the model. In large part due to these two properties, the SVR algorithm will typically outperform the simpler beta regression as a predictive model. However, that performance comes at the cost of interpretability; unlike the beta regressions, the SVR does not have a comparable simple hypothesis test for the significance of each channel to the model. Thus, we use the beta regression to quantify the relationship between neural activity and imagined graded muscle contractions and then switched to SVR to maximize performance for controlling the BCI-FES system.

### Stimulation

The FES system used in this study is the same as described in our previous studies^9, 26^. The system consists of a multi-channel stimulator and a flexible cuff consisting of up to 140 electrodes that is wrapped around the participant’s arm. The center-to-center spacing of the electrodes is 22 mm along the long axis of the forearm and 15 mm in the transverse direction. Electrical stimulation was provided in the form of current-controlled, monophasic rectangular pulses of a 20 Hz pulse rate and 500 µs pulse width. Pulse amplitudes ranged from 0 to 20 mA and were updated every 100 ms. Supplementary Fig. S1 shows a picture of the FES sleeve highlighting the electrodes used in this study. Additional details on the FES system and its calibration can be found in Supplementary Materials.

### Imagined Graded Control of Muscle Contraction and Neural Activity Experiments

**Figure 1:**
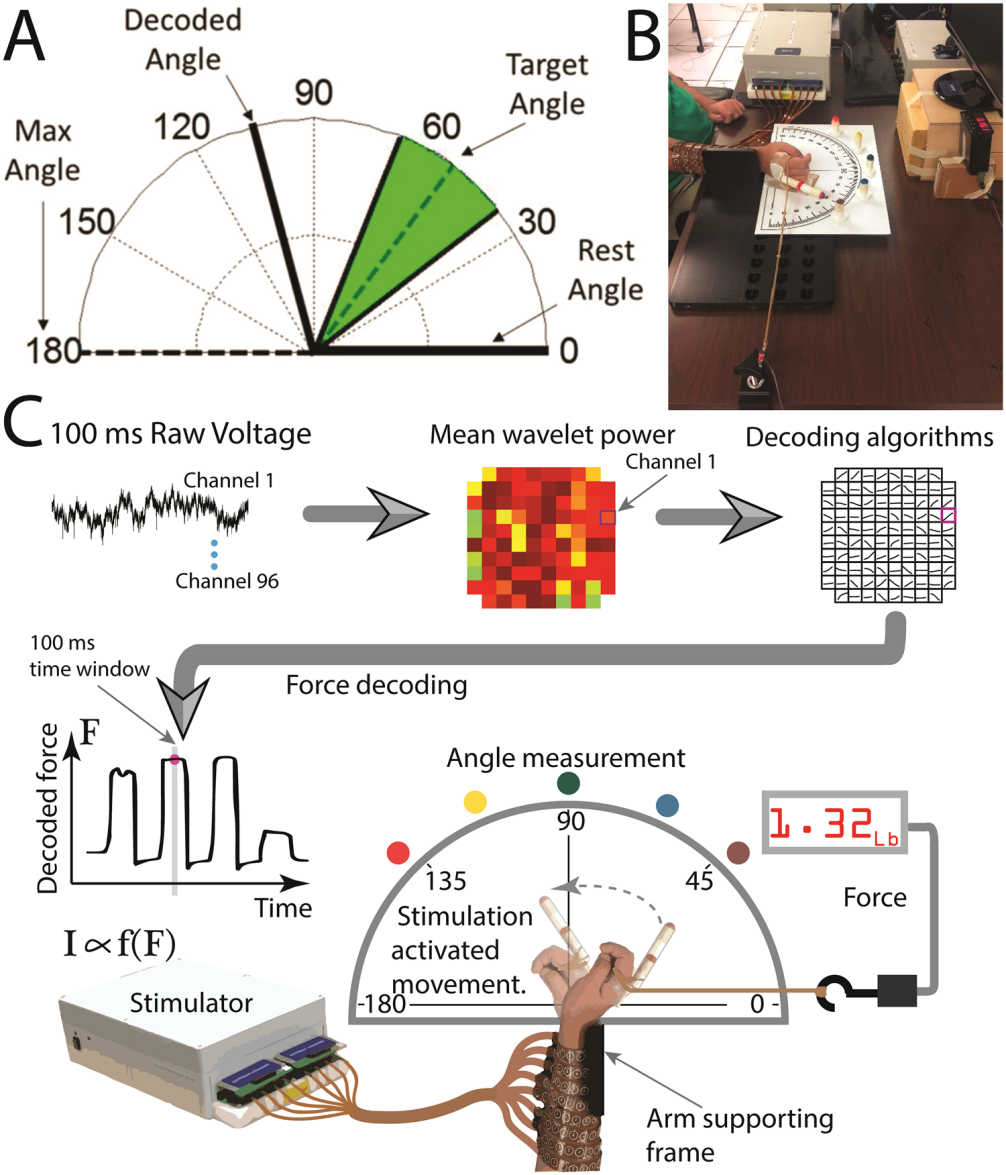
Decoding graded muscle contraction from intracortical activity to control participant’s wrist movement using FES. (**A**) An example screen used for the virtual graded muscle contraction experiment. Potential angles that can be cued range from 0° to 180°. A target angle is cued with a 15° window on either side of the angle (green) and the thick black line tracks the angle decoded from the participant’s modulation in real-time. (**B**) Photograph of the graded control of muscle contraction experiment using the BCI-FES system. (**C**) Flow chart detailing the graded control of muscle contraction experiment where intracortically recorded voltage data are converted to MWP features which are then fed into a decoding algorithm which outputs decoded force, F. The decoded force is then translated into a set of stimulation parameters, I, and the resulting wrist flexion movement is recorded by the load cell and an overhead camera.

To measure the neural activity on individual multielectrode array (MEA) channels to a graded cue, without contamination from stimulation and movement artifacts, we designed an experiment where the participant manipulated a virtual needle to point to four different target angles (52.5°, 90°, 127.5°, and 165°) along a protractor displayed on a computer screen (Fig. 1A). The experiment was designed as a virtual version of physically exerting force via graded muscle contraction (presented in the next section), where increasing cue angles require increased force exertion. Thus, in this experiment, the angle is a proxy measure for imagined force. Neural activity was measured by calculating the Mean Wavelet Power (MWP) feature for each of the 96 channels every 100 ms (see Signal Processing and Decoding and Sharma *et al*.)^26^. MWP has been used successfully in previous studies to control a brain computer interface for a variety of different motor related tasks^9, 27, 28^.

Data was collected in a sequence of 24 consecutive target angles called a block. In each block the participants was presented three repetitions of each of the four non-zero target angles in randomized order. Each target angle was followed by a rest cue (0°). Each block was followed by a short break allowing the participant to rest. Seven blocks of data were collected for a total of 21 replicates of each of the four target angles. A beta regression model was fit using the 96 MWP values as predictor variables and, at most, the three most recent experiments as training data for online decoding of the intended angle. Maximum likelihood estimates were derived for the coefficients as well as hypothesis tests for the significance of each channel to the model. For the first experiment, before a decoder had been trained, the needle was pre-programmed to match the cue. For the remaining six experiments, the output of the model was translated on the screen via the needle, both of which updated every 100 ms. To analyze the relationship between neural modulation and imagined muscle contraction, all seven blocks were combined into one dataset to which the beta regression model was fit offline.

### Graded Control of Muscle Contraction Using BCI-FES Experimental Methods

To investigate whether the BCI-FES system could enable graded control of muscle contraction, we designed a series of experiments where the participant was asked to use the system to grade the flexion of his paralyzed wrist by pointing it at different cued angles along a protractor (Fig. 1B) while working against a resistance. Similar to angle being used as a proxy measure for imagined force as described previously, here we used angle as a proxy measure for exerted force. Due to his injury level, the participant was unable to flex his wrist on his own without the use of the system (see Supplementary Movie S1). When the stimulation was on, the participant had to overcome the resistance of an attached elastic band and grade his muscle contraction to generate the correct amount of force to point at the target angle. A strain gauge based load cell was connected to the other end of the elastic band to measure the force being exerted by the participant as he flexed his wrist.

The participant had a plastic cylinder attached to his hand and was instructed to point the cylinder at one of the five targets. The target angles ranged from 36° (resting state) to 128° (maximum wrist flexion physically possible) covering a range of 92° to exert graded control of wrist flexion. A single block consisted of four non-rest target angles, which were presented three times each for a duration of 5 seconds, each followed by a 5 second rest target cue. The presentation order of the non-rest targets was randomly shuffled to prevent the participant from anticipating the next target. The participant was cued by colors on a computer monitor that matched one of the physical targets positioned on the outside of the protractor (see Fig. 1). A digital camera recording 30 frames per second was placed directly overhead, and the angle at which the participant was pointing and the force exerted were extracted offline from the video data.

For scoring each non-rest cue, the participant had a 6.5 s window (5 s cue plus 1.5 s grace period), while each rest cue had a 3.5 s window (5 s cue minus preceding 1.5 s grace period). The 1.5 s grace period was added to the end of each movement cue to account for system lag, reaction time, and the variable time it took the participant to physically move his wrist to the different positions. For both types of cues, success was defined by achieving an angle within 15° of the target and staying within 15° of the target continuously for 2 s. The 15° tolerance was chosen because it was approximately three widths of the pointing cylinder, and the duration criteria ensured that the participant sustained the graded flexion against the resistance of the elastic band and did not receive credit for briefly moving through the target area.

Prior to the first block, stimulation parameters were calibrated (see Supplementary Materials for details). During the first block, the participant received stimulation corresponding to the cue since no neural decoder had been built yet. The stimulation allowed the algorithms to learn from data acquired while the stimulation system was on and the participant’s hand was moving. Using data from the first block, an initial decoder was trained. Decoding was performed using the same MWP features as before but using the SVR algorithm. In all subsequent blocks, stimulation was exclusively determined by the output of the decoders interpreting the participant’s neural modulation in real time. Starting with the second block, the participant was controlling the system volitionally. The second block provided us with the first data where the participant was controlling the stimulation system. This was followed by three training blocks that were used to build the decoder for the remainder of the blocks. The training blocks were followed by six test blocks. The first four test blocks were set up identically to the training. In the final two blocks, the participant was challenged with angles he had not attempted previously. A summary of all the blocks, their target angles, and their purpose is presented in Supplementary Table S1.

## Results

### Imagined Graded Control of Muscle Contraction and Neural Activity

**Figure 2:**
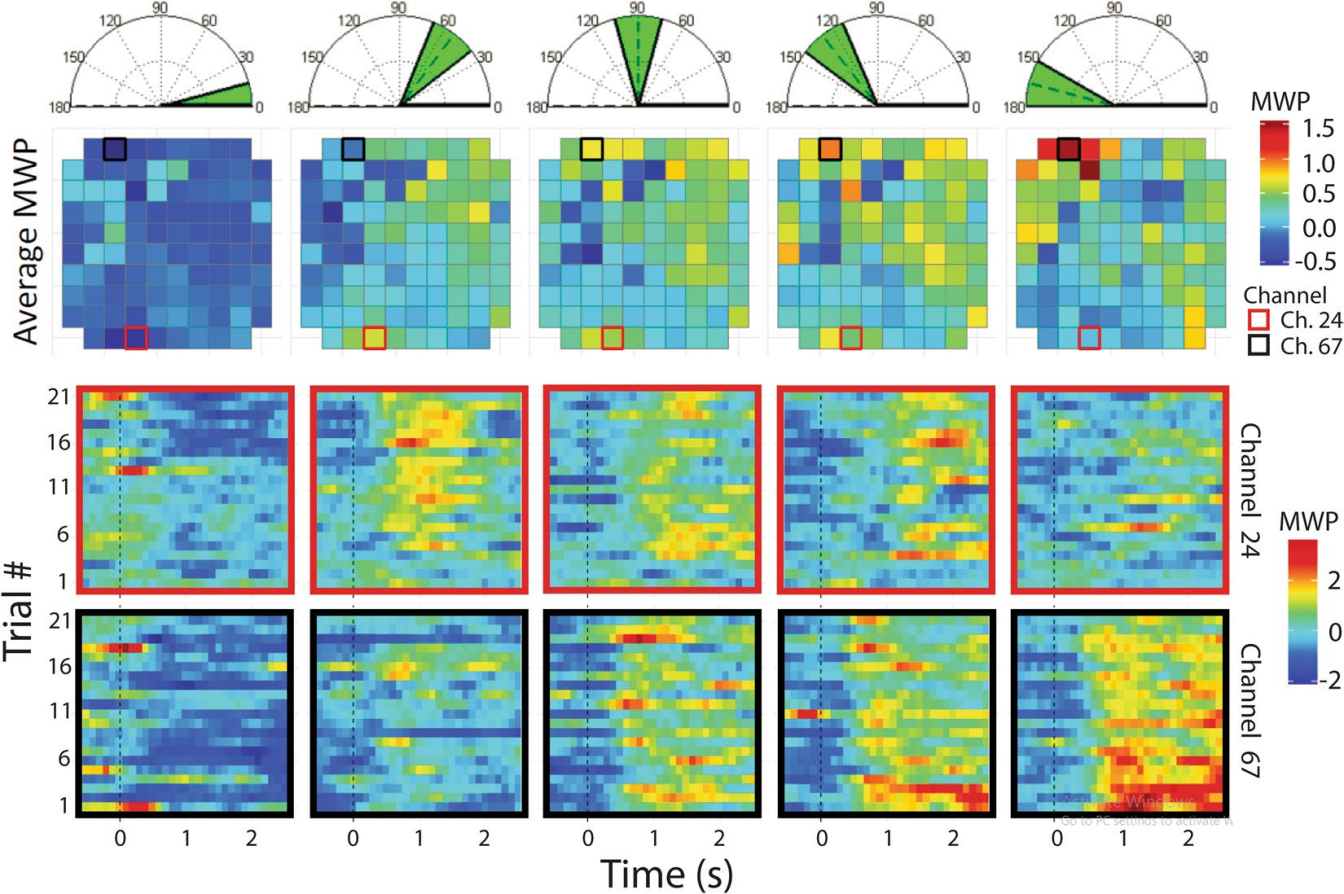
Imagined graded muscle contractions modulates neural activity in M1. The top row shows the angle cue that was presented visually to the participant. Each column corresponds to the cue from the top row. The second row shows the mean MWP superimposed on the layout of the MEA. The MWP is averaged over each trial from 0.5 s after cue presentation to 2.5 s after cue presentation. The third row shows the temporal evolution of MWP for each of the 21 trials (for the rest cues we show the first 21 trials) for Channel 24 from 0.5 s before the cue until 2.5 s after the cue. The dashed line at 0 s represents the time of cue presentation. Channel 24 shows a pattern of low MWP for the rest angle, high MWP activity at the lower non-zero angles and decreasing MWP activity as the angles increase. The last row follows the same format as above but represents Channel 67, which shows increasing MWP activity with increased angles.

**Figure 3:**
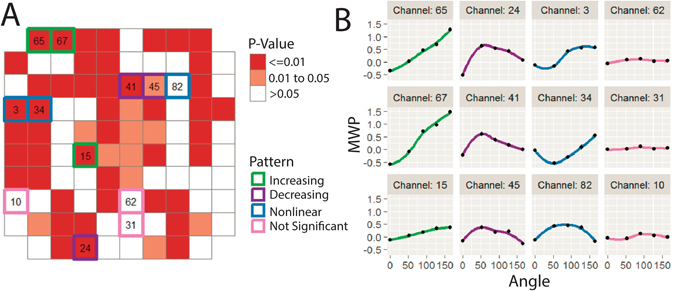
Neural modulation patterns with imagined graded muscle contractions. (**A**) The p-values from the beta regression are overlaid over the physical layout of the microelectrode array (MEA). Selected individual channels (highlighted in A) demonstrate a range of neural modulation trends in response to the cued angles. (**B**) Each column shows selected individual channels where the average MWP (1) increased with increasing cue angles (green), (2) decreased with increasing non-zero cue angles (purple), (3) showed a non-linear relationship with cue angle (blue) and (4) did not show significant response to the cue angles (pink). The average MWP values are calculated the same as in Fig. 2.

We first measured the changes in neural activity resulting from the participant imagining flexing his wrist to point at four different target angles. Offline, a beta regression model was fit to the neural data to predict the imagined angle. The beta regression identified that several channels contained useful information for decoding the target angle; 56 of the 96 channels in the electrode array contributed significantly (*p* < 0.05) to the beta regression model. In addition, 45 of those 56 channels were highly significant (*p* < 0.01). A full summary of the regression parameters and their significance is provided in Supplementary Table S2. To investigate further, the neural activity of each channel over time was aggregated over all trials for each of the four target angles and rest. On several channels, the neural activity as represented by the MWP values, tracked proportionally to the cued angle. Two striking examples were observed on Channels 67 and 24 as seen in Fig. 2. On channel 67, the MWP increased as the cued angle increased as seem in Fig. 3. The increases in MWP from the 52.5° target to 90° target and from the 127.5° target to the 165° target were statistically significant (*p* values are 0.008 and 0.033, respectively, via permutation test); the increase in MWP from the targets 0° to 52.5° and from 90° to 127.5° were not statistically significant (*p* values are 0.050 and 0.177, respectively). Channel 67 also had the largest coefficient in the regression model (*p* < 0.001), indicating that output from this channel greatly influences the predictions from the beta regression model. The inverse relationship between MWP and cued angle was observed in channel 24 (Figs 2 and 3). Excluding rest, MWP values decreased as the cued angle increased. However, unlike Channel 67, the relationship between MWP and the cued angle is not linear. MWP increased between the 0° target and the 52.5° target (*p* < 0.001) but decreased with increasing non-zero target angles (52.5° to 90°, 90° to 127.5°, and 127.5° to 165°; *p* values were 0.083, 0.360, and 0.002, respectively). While the change in MWP among the middle angles—52.5°, 90°, and 127.5°—are not statistically significantly different from one another, they all show the same trend of MWP being significantly higher when compared to 0° (all *p*-values < 0.001) and significantly lower compared to the largest angle of 165° (*p* < 0.001, 0.001, and 0.002, respectively). Several other channels showed distinct relationships between neural activity and imagined force; examples are shown in Fig. 3.

While the beta regression model is useful for identifying individual channels where neural activity was strongly correlated with the cued angle, it assumes a monotonic relationship between MWP and angle and, thus, cannot adequately capture more complex relationships like that observed for channel 24. Therefore, we explored a more sophisticated algorithm that handles complex relationships better in exchange for a loss in interpretability: the non-linear support vector regression (SVR) algorithm^29^. Additionally, the SVR algorithm utilizes regularization, which minimizes the effect of non-informative channels (recall 40 of the 96 channels were not significant). Compared to the beta regression, we saw a significant improvement with SVR in the predictive accuracy which went from 27.2° to 21.7° median absolute error (*p* < 0.001). These results indicate that there are multiple channels that modulate corresponding to the imagined graded muscle contraction cues. In the next experiment, this modulation was decoded in real time to control stimulation, enabling our study participant to grade the contraction of his paralyzed muscles.

### Graded Control of Muscle Contraction Using BCI-FES System

**Figure 4:**
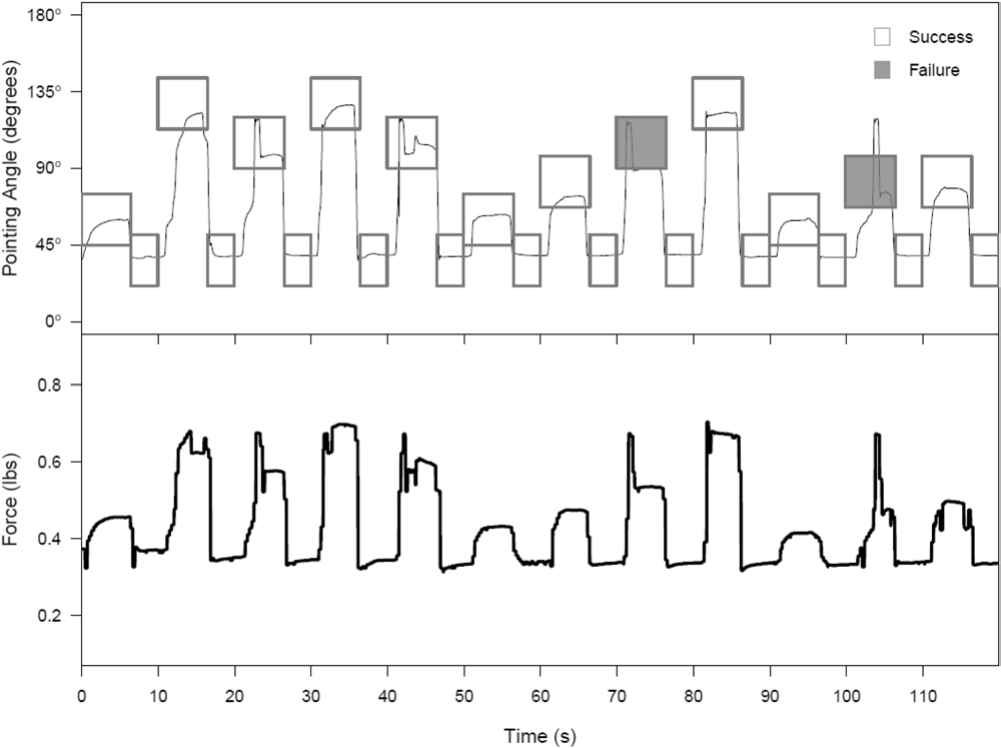
The participant generated graded muscle contractions to pull against resistance and point at various target angles. The top figure shows the cued angle (rectangles) and the angle at which the participant was pointing (solid black line) for the first training block. If the participant was successful for a given cue (within 15° of the cue for 2 s continuously), the rectangle is clear; if he failed the cue, it is shaded gray. The bottom figure shows the force measurement in pounds as measured by the load cell aligned in time for the same block. The force exerted closely tracks the wrist flexion as measured by the pointing angle (correlation coefficient 0.933 ± 0.006).

In this experiment, the participant manipulated the muscle contraction of his own wrist using the BCI-FES system to match different target angles. The participant successfully hit the target angles in the set of three training blocks at a rate of 90.3 ± 3.5% (mean ± standard error), where success was defined as maintaining position within a ± 15° window around the target cue angle for at least 2 continuous seconds. For example, the participant was successful on 22 of the 24 cues shown during the first training block (Fig. 4 and Supplementary Movie S2). On both failed cues, he initiated movement and pointed his wrist in the general direction of the cue but was not able to sustain in the target window continuously for the full 2 seconds. The bottom panel of the plot shows the generated force as measured by the load cell. The force generated closely tracks with the angle at which the participant was pointing (correlation coefficient 0.933 ± 0.006), indicating that the angle is a good proxy measure for force exerted (see also Supplementary Fig. S2). The results for the other two training blocks were similar and are summarized in Table 1. The neural decoder continued to evolve during these three blocks based on the data collected from the preceding blocks, as explained in Methods. After the third training block, the decoder was fixed and used for all remaining test blocks.

**Table 1:** Summary of Participant Accuracy Over the Three Training Blocks.

Description	Block #	Rest Angle (36°)	Angle A (60°)	Angle B (82°)	Angle C (105°)	Max Angle (128°)	Total Correct	Accuracy
Training 1	3	12/12	3/3	2/3	2/3	3/3	22/24	91.7%
Training 2	4	12/12	3/3	3/3	1/3	3/3	22/24	91.7%
Training 3	5	12/12	3/3	2/3	1/3	3/3	21/24	87.5%
Total	3-5	36/36	9/9	7/9	4/9	9/9	65/72	90.3%

The participant’s performance dropped significantly for the next three test blocks (Table 2). The participant was successful on all rest and 60° angle cues but was only successful on 3 of 27 cues for the three highest angles (82°, 105°, and 128°). Overall, his performance in achieving the cues in the test blocks was 66.7 ± 5.5%. This decline in performance could be due to a number of factors, including physical and mental fatigue and/or system limitations. Therefore, in order to better understand the probable cause in this decline in performance, we calculated the difference between the neural decoded angles and the FES-enabled physically achieved wrist angles. We defined this as the calibration difference distribution (CDD). Ideally, the FES should provide the correct amount of stimulation to flex the wrist to the exact angle predicted by the decoder. Under that ideal condition, the difference would be zero. Supplementary Fig. S3 shows the CDD for each block as a boxplot. It is very apparent that while, on average, the differences are all close to zero, the interquartile range (IQR) increased from an average of 8.6° during the first three training blocks to 26.6° during the next three test blocks, indicating that during the test blocks there were sizable mismatches between the neutrally decoded angle that the stimulation was supposed to reach and the actual wrist angle achieved through FES. In addition, our observation that the errors occurred predominantly for the higher angles suggest the culprit is muscle fatigue that hindered the participant’s ability to achieve the desired wrist angle despite the correct neurally decoded angle. Recalibrating the stimulation had an immediate effect on overall performance in the subsequent test block; the IQR dropped to 10.0 degrees (Supplementary Fig. S3) and the participant’s performance to hit the target angles improved to 87.5 ± 6.8% for Test Block 4. A comparison of the three tests blocks prior to stimulation recalibration and the three test blocks after can be found in Table 2.

**Table 2:** Summary of Participant Accuracy Over Test Blocks.

Description	Block #	Rest Angle (36°)	Angle A	Angle B	Angle C	Max Angle (128°)	Total Correct	Accuracy
Test 1	6	12/12	3/3 (60°)	0/3 (82°)	1/3 (105°)	0/3	16/24	66.7%
Test 2	7	12/12	3/3 (60°)	0/3 (82°)	0/3 (105°)	1/3	16/24	66.7%
Test 3	8	12/12	3/3 (60°)	0/3 (82°)	0/3 (105°)	1/3	16/24	66.7%
Test 4	9	12/12	3/3 (60°)	2/3 (82°)	3/3 (105°)	1/3	21/24	87.5%
Generalization Test 1	10	12/12	3/3 (57°)	1/3 (76°)	3/3 (111°)	3/3	22/24	91.7%
Generalization Test 2	11	12/12	3/3 (71°)	2/3 (90°)	2/3 (114°)	2/3	21/24	87.5%
Test pre-recalibration	6–8	36/36	9/9	0/12	1/12	2/12	48/72	66.7%
Test post-recalibration	9–11	36/36	9/9	5/9	8/9	6/9	64/72	88.9%
Generalization Blocks	10–11	24/24	6/6	3/6	5/6	5/6	43/48	89.6%

Stimulation parameters were recalibrated between the third and fourth test block. Angles A, B, and C were at 60°, 82°, and 105° respectively for blocks 6–9 and were moved to angles not previously attempted—57°,76°, and 111° for the first generalization test block (Block 10) and 71°, 90°, and 114° for the second generalization test block (Block 11).

**Figure 5:**
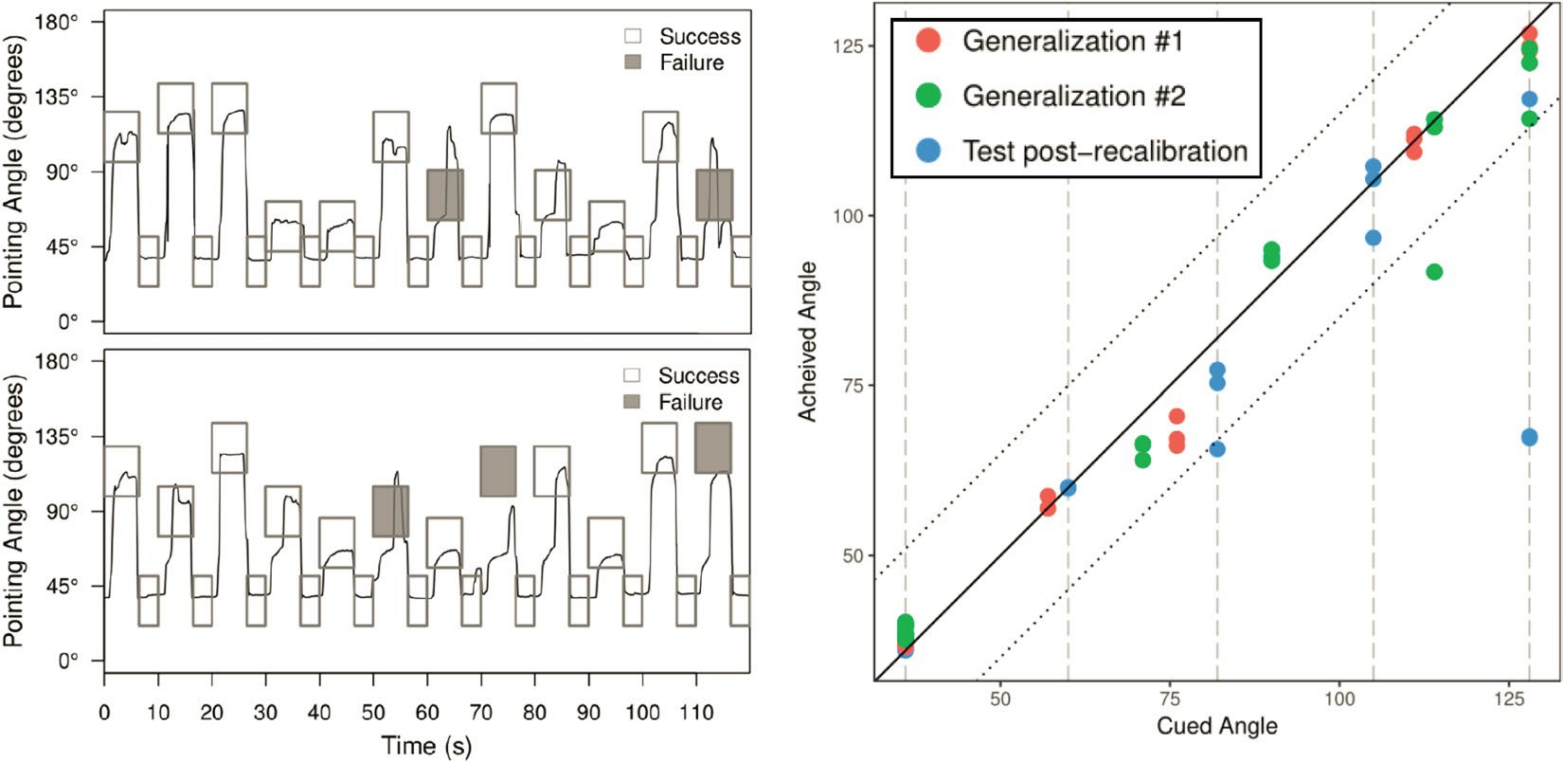
(Left) Participant generates force to match angles that were not used during training in Generalization Blocks 1 (top left) and 2 (bottom left). For these two blocks, the three middle angles were moved to positions the participant had never attempted before. The participant was successful on 22 of 24 cues for the first generalization block and 21 of 24 for generalization block 2. In both blocks, he was successful on 7 of the 9 cues for new angles. (Right) For each cue in the generalization blocks as well as Test Block 4 (post-recalibration) the 2 s where the average achieved angle was closest to the cued angles was extracted and the average achieved angle over that 2 s is plotted against the cued angle. The colors denote the different blocks. The diagonal black line indicates the line of perfect performance where the achieved angle is exactly the cued angle; the upper and lower dotted black lines show the 15° tolerance bands around the middle line. The vertical dashed lines indicate angles used for the Training and Test Blocks 1-4. The plot demonstrates that the participant was not only in the tolerance window during the generalization blocks but also could point to the new angles with similar precision to the angles used during training.

We also investigated whether the participant could accurately grade generated wrist force to a new set of angles and not just the five angles that were used in the preceding blocks. We constructed two additional test blocks, called generalization blocks, where the rest and maximum angle cues were unchanged but the three intermediate angle cues were moved along the protractor to different angles not previously attempted. Across the two generalization blocks, the participant achieved an accuracy of 89.6 ± 4.4% including successes on 14 out of a possible 18 cues for angles that he had not attempted previously (see Table 2, Fig. 5, Supplementary Fig. S4 and Supplementary Movie S3). Thus, our system provides the first demonstration of *continuous* volitional control of graded muscle activation in a paralyzed human.

## Discussion

In this study, we have shown that neurons in M1 carry information that can be reliably decoded to allow a human with tetraplegia to regain volitional control of graded muscle contraction with the use of our BCI-FES system. Graded movements are crucial for performing fine motor tasks such as grasping delicate objects with the appropriate amount of force (for example, grasping a paper cup full of water) or manipulating objects of varying weights. With the use of our BCI-FES system, the participant was able to generate a continuum of force levels between 0.3 and 0.7 lbs in order to match his wrist position to the various cued angles (see Supplementary Fig. S4). This represents an important advancement toward allowing SCI patients to regain more natural control of hand usage.

In the virtual experiment, we quantified the neural activity related to imagined graded muscle contraction, without contamination from stimulation and movement artifacts, by using a virtual approximation of the experiment for graded control of muscle contraction. We initially used a simple linear beta regression model-based decoder in the system. This was done to investigate whether certain channels from the MEA modulate reliably with imagined muscle contraction. We observed that on some channels, neural activity increases monotonically with cue angle (Fig. 3). The monotonic increase in neural activity with cue angle we observed is consistent with human fMRI studies, which showed that increasing force production is associated with linear increases in the BOLD signal of M1^17–19^. However, we also observed channels that showed decreasing levels of modulation with increasing cue angles as well as several different non-linear relationships (Fig. 3). These channels may also contain positional information, which, in addition to force information, is known to be represented in M1^30^. As such, we do not attempt to disambiguate the two as graded muscle contraction movements contain both position and force components. Regardless, our results strongly imply that, at finer scales, the relationship between neural activity and graded muscle contraction may be more complex than a simple positive correlation. This has significant implications for decoding this information from neural activity, as decoding algorithms utilized should be able to accommodate complex nonlinear relationships between modulation and graded muscle contraction. Although the beta regression model provided important insights into the underlying neural modulation, its simplicity limits its practical use in a BCI-FES system. We therefore switched to a SVR algorithm (which can accommodate nonlinear relationships) to provide a more robust control signal to the FES system for stimulation of wrist flexion. Using the system, the participant was able to volitionally flex his wrist against resistance across a continuous 92° range of motion.

Our results extend previous work on graded muscle contraction with BCI-FES systems^6, 7^. These studies used monkeys who were temporarily chemically paralyzed. It is difficult to interpret the applicability of these system to human SCI patients, as chemical paralysis is known to bypass several complications stemming from spinal cord injuries^31, 32^. We have shown that our BCI-FES system works for a human SCI patient who has not been able to move his hand and wrist on his own for over 6 years since his injury. Using the system, he became the first human who was able to use his thoughts to grade and sustain flexion of his own paralyzed wrist. He was able to reliably point at the target angles with an average success rate of 88.9 ± 3.7% post-stimulus recalibration. Additionally, the generalization test blocks results demonstrate that, using the system, he was able to volitionally control his own graded muscle contraction across a continuous range of angles, including angles that were not included in the training data (Fig. 5), thus providing the first demonstration of truly continuous control of graded muscle activation in a paralyzed human. In the future, our approach could be expanded to enable a user to volitionally deliver an appropriate level of force when applying grips to delicate objects. Restoring the ability to grade muscle contractions in paralyzed individuals will allow them to gain higher levels of functional independence and has important implications for the development of new methods for restoring movement in people living with paralysis from SCI, stroke, brain injury, motor neuron disease, and other debilitating conditions.

## Electronic supplementary material

Supplementary Materials

Movie S1

Movie S2

Movie S3
