# Supplementary material for: Neuroprosthetic-enabled control of graded arm muscle contraction in a paralyzed human
Source: Sci Rep. 2017 Aug 21;7:8386. doi: 10.1038/s41598-017-08120-9 (PMC5567199; doi:10.1038/s41598-017-08120-9)
Supplement: Supplementary file 1 — Supplementary Materials [file 41598_2017_8120_MOESM1_ESM.pdf]

Supplementary Materials for

Neuroprosthetic-enabled control of graded arm muscle contraction in a paralyzed human

David A. Friedenberg PhD[1,*], Michael A. Schwemmer PhD[1], Andrew J. Landgraf PhD[1], Nicholas V. Annetta MS[2], Marcia A. Bockbrader MD PhD[3,4], Chad E. Bouton MS[2]†, Mingming Zhang PhD[2], Ali R. Rezai MD[3,5], W. Jerry Mysiw MD[3,4], Herbert S. Bresler PhD[2], Gaurav Sharma PhD[2]

correspondence to: friedenbergd@battelle.org

This file includes:

Supplementary Methods
Supplementary References
Figs. S1 to S4
Tables S1 to S2
Captions for Movies S1 to S3

Other Supplementary Materials for this manuscript includes the following:

Movies S1 to S3

Supplementary Methods

Stimulation Parameters and Calibration

The stimulation intensity and spatial pattern were determined by using a trial-and-error method. After the operator determined the pattern needed in order to evoke the wrist flexion with the elastic band pulling on the hand, the stimulation intensity was captured at three levels producing: (1) wrist flection that just started to move the wrist from the rest position (low); (2) wrist flexion that pointed the hand straight ahead (medium); and (3) flexing of the wrist to full deflection (high). The three levels of deflection were all visually determined by the operator. The decoder output score (after the stabilizing filter), r_{filt}, was then used to set the stimulation level, $I_p(r_{\text{filt}})$, for each low-side electrode during real-time decoding. A piecewise linear function was used to in order to interpolate between the three calibrated stimulation intensities as follows:

$$I_p(r_{\text{filt}}) = \begin{cases} 0, & r_{\text{filt}} < 0.2 \\ c\,[r_{\text{filt}}\,(m - l) + 0.5l - 0.2m]/0.3, & 0.2 \le r_{\text{filt}} < 0.5 \\ c\,[r_{\text{filt}}\,(1 - m) + 0.71m - 0.5]/0.21, & 0.5 \le r_{\text{filt}} < 0.71 \\ c, & r_{\text{filt}} \ge 0.71 \end{cases}$$

where c is the pulse current calibrated at maximum deflection in milliamps and where l and m are multipliers to that, calibrated to low and medium deflection, respectively, in the range of 0 to 1. The stimulation current level $I_p(r_{\text{filt}})$ is in terms of pulse amplitude in milliamps. The thresholds of 0.2, 0.5, and 0.71 were derived empirically and were used to compensate for the nonlinear response of the muscles to surface stimulation.

During the Graded Control of Muscle Contraction Using BCI-FES System task, the stimulation patterns used the electrodes shown in Fig. S1. The low side electrodes were initially calibrated to pulse amplitudes of 10.74 mA. These were later recalibrated to 14.00 mA between Test Blocks 3 and 4. The low and medium multipliers were set to 0.30 and 0.42, respectively, for both calibrations.

Smoothing Decoder Output

To facilitate smooth movement, the output of the decoder was filtered. The filter allowed the system to respond quickly to strong, fast changes in the decoder output, but maintain a steady output when the decoder output was not changing significantly.

During every 100ms processing loop, after the decoder output, r, is calculated, the filter windows are updated, and finally the filtered output, r_{filt}, is calculated using the windows as follows:

If $r < \mathrm{b_{lo}}$, set $\mathrm{b_{lo}} = r$ and $\mathrm{b_{hi}} = r + \mathrm{W}$.
Else if $r > \mathrm{b_{hi}}$, set $\mathrm{b_{hi}} = r$ and $\mathrm{b_{lo}} = r - \mathrm{W}$.

$$r_{\text{filt}} = \frac{\mathrm{b_{hi}} + \mathrm{b_{lo}}}{2}$$

where b_{hi} and b_{lo} are the upper and lower bounds of the filter window and W is the size of the filter window ($W = 0.2$ for these experiments).

Estimating wrist position for the Imagined Graded Control of Muscle Contraction Experiment

During the Imagined Graded Control of Muscle Contraction task, the virtual needle was modeled after the wrist to give the subject similar position feedback to the Graded Control of Muscle Contraction Using BCI-FES System task. A simple model was created to estimate wrist position.

The net torque on the wrist, τ, was empirically derived to be

$$\tau = \begin{cases} \tau_{contract} - \mu\omega{\theta_i}^2 - A{\theta_i}^3, & \omega \leq 0 \\ \tau_{contract} - \frac{\mu\omega{\theta_i}^2}{2} - A{\theta_i}^3, & \omega > 0 \end{cases}$$

where$\tau_{contract}$ is the torque on the wrist due to the stimulation induced muscle contraction, calculated based on scaling the decoder output score; μ is the empirically derived damping/friction coefficient; ω is the angular velocity of the wrist; θ_i is the initial angle of deflection of the wrist; and A is an empirically derived constant.

From torque, the final angular position of the wrist, θ_f, can be calculated by

$$\alpha = \frac{\tau}{ml^2}$$

$$\omega_f = \omega_i + \alpha\Delta t$$

$$\theta_f = \theta_i + \omega_i\Delta t + \frac{1}{2}\alpha(\Delta t)^2 = \theta_i + \frac{\omega_o + \omega_f}{2}\Delta t$$

where α is the angular acceleration of the wrist; l is the distance between the wrist and the point of contact of the rubber band and the hand; m is the empirically derived mass of the hand; ω_f is the final angular velocity of the wrist; ω_i is the initial angular velocity of the wrist; and Δt is the change in time.

Permutation Test

Permutation tests were run for each channel to test whether the peak MWP was different between the cued angles. For each channel and cued angle, a LOESS (1) smooth curve was fit to the MWP as a function of time since the start of the cue, as shown in Fig. 3. The peaks of the LOESS curves for each of the cued angles was recorded. In order to avoid finding peaks that are the result of high MWP from the previous cue, we only looked for the peak between 0.5 s and 2.5 s. Random permutations were generated in order to create a null distribution where the peaks are expected to be the same. For each permutation, the cued angles were randomly swapped, the LOESS curves were fit again for each cued angle, and the peaks of the LOESS curves for each angle were recorded; 5,000 random permutations were generated and their associated peaks were recorded. Finally, the difference between the peaks at different cued angles of the LOESS curves from the actual data was compared to the differences in the permuted data. For example, for the null hypothesis that angle A is less than or equal to angle B, if the actual difference in the peak MWP from angle A and angle B is larger than 950 of the permuted

differences, then the p-value is 0.050.

Comparison of Beta Regression and SVR

To compare beta regression and SVR, we trained both algorithms on the first five blocks of the imagined graded control of muscle contraction experiment and predicted the response on the final two blocks. The response was lagged by 0.8 s to allow for reaction time similar to Bouton et al. (2). The Wilcoxon rank sum test was used to test the null hypothesis that the median of the predicted absolute errors is larger for SVR than for beta regression. The null hypothesis was rejected with a p-value less than 0.001, giving strong evidence that SVR has a lower median absolute error than beta regression.

Video Evaluation Metrics

A digital camera was positioned directly over the participant’s hand and recorded HD video at 30 frames per second during the experiment. The video was aligned in time with the cues using an audio cue prior to the beginning of the experiment. The two colored cots placed on the plastic cylinder were used to locate the cylinder in space. A line was drawn from the center pixels of two colors, and the angle at which that line intersected the protractor was recorded as the observed angle. When the two points cannot be located due to motion blur, the last valid observed angle is used as a substitute. We created a 15° target window on either side of the target angle and measured the longest continuous block of time that the observed angle was sustained within the target window. This metric is calculated for the duration of the 5 s duration cue plus an additional 1.5 s buffer for non-rest cues to account for reaction time and the variable time it takes for the participant to physically move from rest to the target angle. We categorize a cued movement as successful if the participant sustained position in the target window for at least 2 s. Additionally, the exerted force was read off of the load cell using an object character recognition algorithm. A median filter with a window size of three was applied to the load cell values to correct the occasionally misidentified character.

Supplementary References

1. W. S. Cleveland, E. Grosse, W. M. Shyu, Local regression models. *Statistical models in S* **2**, 309 (1992).
2. C. E. Bouton, A. Shaikhouni, N. V. Annetta, M. A. Bockbrader, D. A. Friedenberg, D. M. Nielson, G. Sharma, P. B. Sederberg, B. C. Glenn, W. J. Mysiw, A. G. Morgan, M. Deogaonkar, A. R. Rezai, Restoring cortical control of functional movement in a human with quadriplegia. *Nature* **533**, 247 (May 12, 2016).

Fig. S1.

Electrode cuff wrapped around forearm of the participant. Electrodes marked in red show anode and electrodes marked in black show cathode for stimulation.

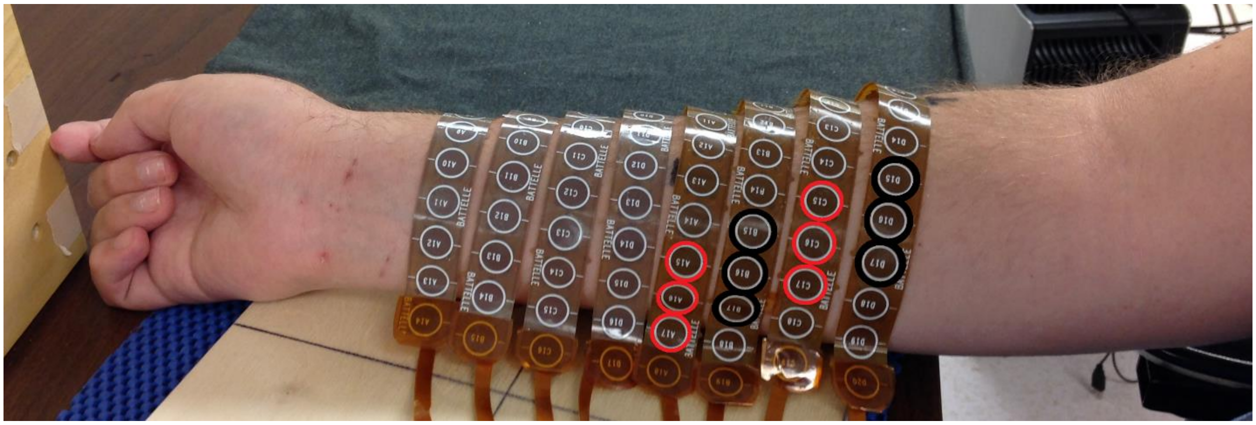
BATTELLE

Fig. S2.

Cued angle is a good proxy for exerted force. Force exerted is plotted as a function of cued angle for both the training and test post-recalibration trials. The solid dots represent successes while the crosses represent times when the patient failed to match the cued angle. The two lines are the best-fit linear models for the successful training and test post-recalibration data points. The R^2 value for both lines is 0.98, indicating that when the participant is successful, force is strongly linearly related to the cued angle.

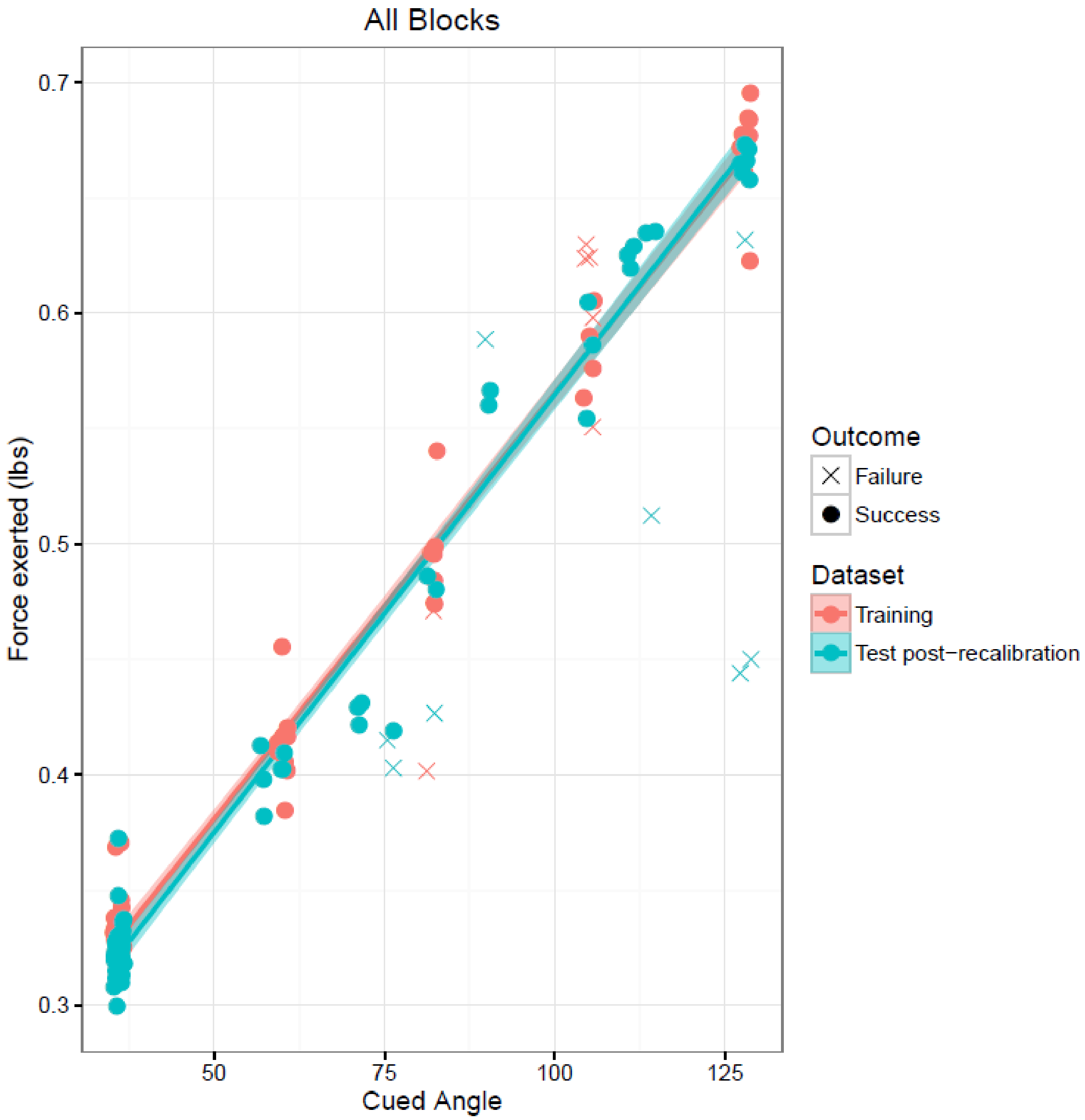

Fig. S3.

Stimulation recalibration counteracts decrease in performance. Boxplots show the calibration difference distribution (CCD) for each block. The spread greatly increases starting with Test Block 1, which we believe is due to muscle fatigue, causing the same amount of stimulation to generate less muscle contraction. The stimulation parameters were recalibrated between Test Blocks 3 and 4 and led to a sizable decrease in the spread of CCD and a corresponding improvement in performance.

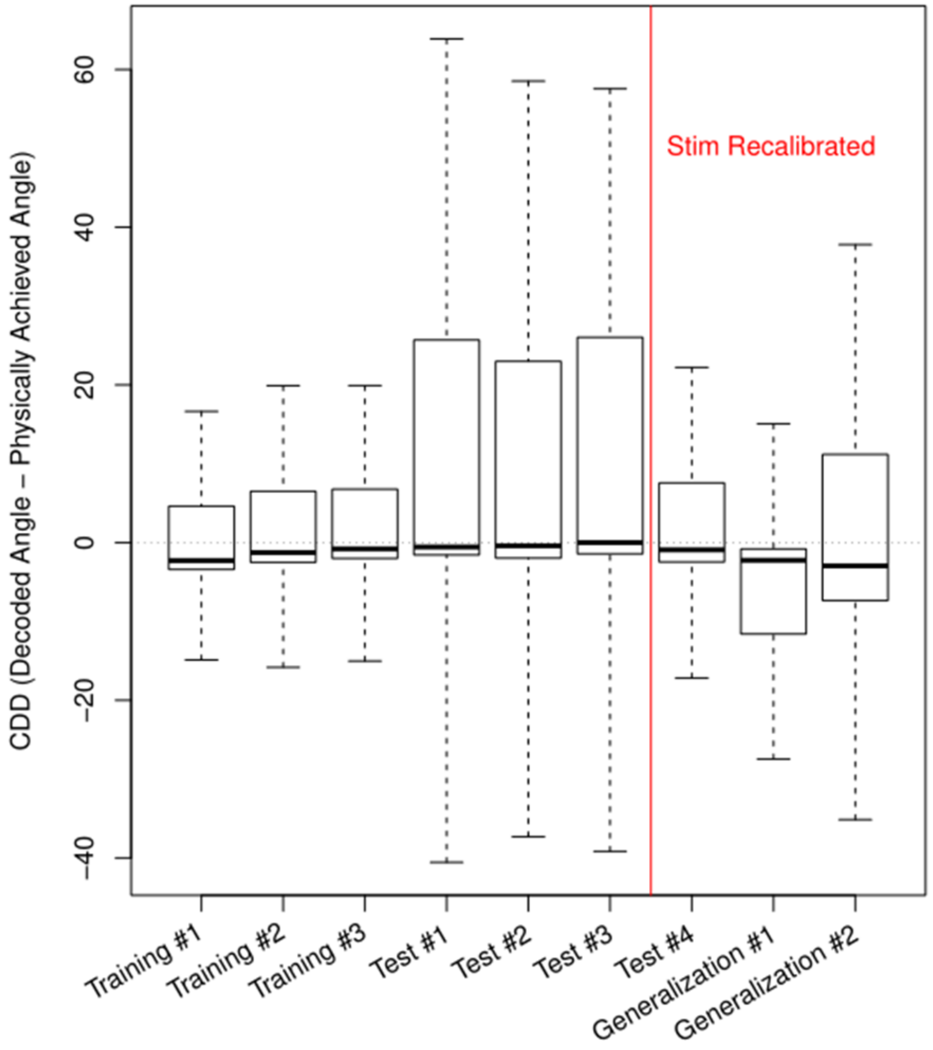

Fig. S4.

For each cue, the 2 s where the average achieved angle was closest to the cued angles was extracted and the average achieved angle over that period is plotted against the cued angle. The colors show dataset groupings with linear regression lines and corresponding confidence bands. The middle dotted black line indicates the line of perfect performance where the achieved angle is exactly the cued angle; the upper and lower dotted black lines show the 15° tolerance bands of the target window.

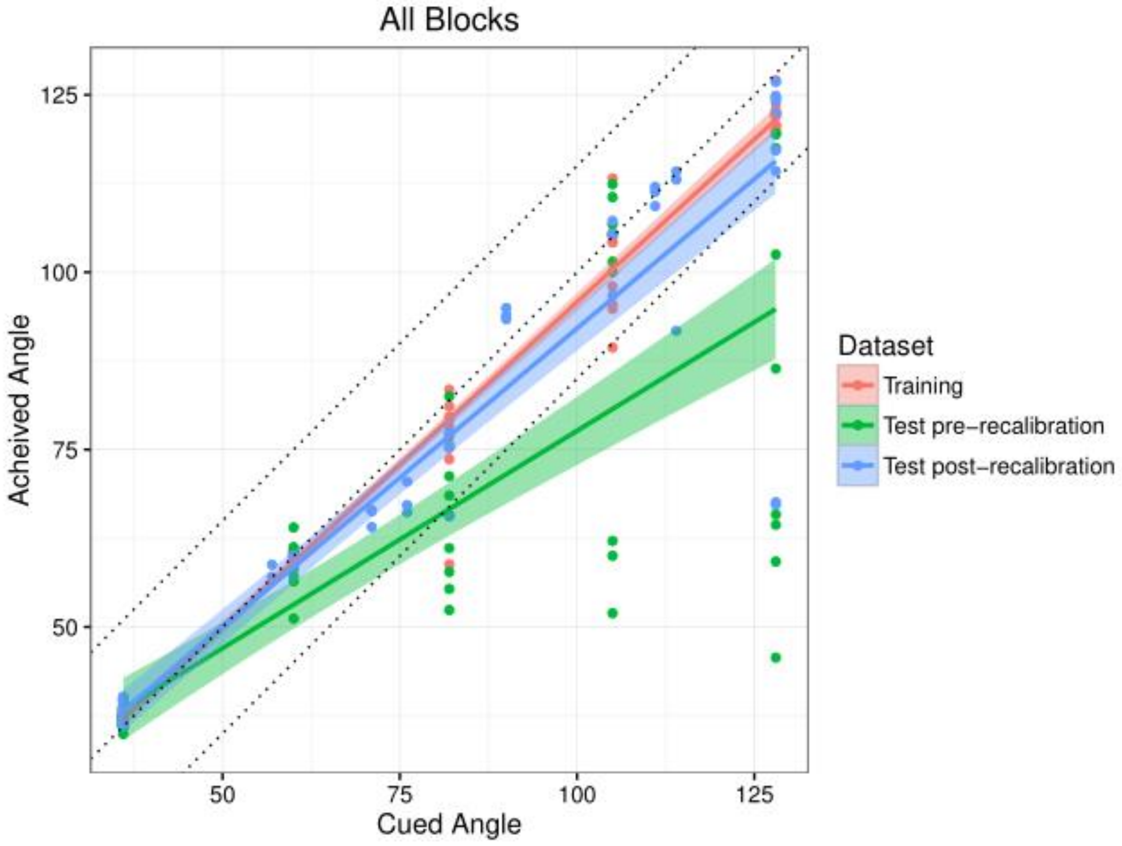

Tables

Table S1.

Summary of FES-enabled force experiment. Stimulation parameters were recalibrated prior to Test Block 4.

Block Number	Block Name	Angles	Decoder Training Data
1	Setup 1	[36 60 82 105 128]	No Decoder
2	Setup 2	[36 60 82 105 128]	Setup 1
3	Train 1	[36 60 82 105 128]	Setup 1-2
4	Train 2	[36 60 82 105 128]	Setup 1-2 Train 1
5	Train 3	[36 60 82 105 128]	Setup 2 Train 1-2
6	Test 1	[36 60 82 105 128]	Train 1-3
7	Test 2	[36 60 82 105 128]	Train 1-3
8	Test 3	[36 60 82 105 128]	Train 1-3
9	Test 4	[36 60 82 105 128]	Train 1-3
10	Generalization Test 1	[36 57 76 111 128]	Train 1-3
11	Generalization Test 2	[36 71 90 114 128]	Train 1-3

Table S2.

Summary of beta regression model parameters.

Channel #	Estimate	Std Err	z-stat	p-value
Intercept	-1.04607	0.01307	-80.0357	0
1	-0.03664	0.02226	-1.64616	0.09973
2	0.11518	0.02352	4.89741	0
3	-0.10637	0.02416	-4.40314	0.00001
4	0.08947	0.02259	3.96023	0.00007
5	-0.03882	0.02034	-1.9086	0.05631
6	0.10008	0.02572	3.89042	0.0001
7	0.19409	0.01815	10.6912	0
8	-0.0775	0.03	-2.58341	0.00978
9	-0.2104	0.01987	-10.5897	0
10	0.00616	0.02816	0.21865	0.82692
11	0.09806	0.03129	3.1339	0.00173
12	-0.05437	0.03029	-1.79512	0.07263
13	0.05376	0.02859	1.88029	0.06007
14	0.00938	0.03272	0.28678	0.77428
15	-0.06346	0.02152	-2.94924	0.00319
16	-0.08281	0.03288	-2.51813	0.0118
17	0.02952	0.01677	1.76014	0.07838
18	0.00475	0.03244	0.1465	0.88353
19	0.0476	0.02933	1.62287	0.10462
20	-0.11278	0.02406	-4.68761	0

Channel #	Estimate	Std Err	z-stat	p-value
49	-0.01209	0.03415	-0.35405	0.7233
50	-0.05615	0.02473	-2.27088	0.02315
51	-0.05636	0.02603	-2.16546	0.03035
52	-0.06182	0.0247	-2.50285	0.01232
53	0.01412	0.03354	0.42107	0.6737
54	0.15313	0.02333	6.56469	0
55	0.0016	0.02779	0.05757	0.95409
56	0.087	0.02805	3.10113	0.00193
57	-0.02515	0.02405	-1.04573	0.29569
58	0.03337	0.02031	1.64291	0.1004
59	0.08445	0.02106	4.00985	0.00006
60	0.08509	0.02736	3.10987	0.00187
61	-0.0963	0.02371	-4.06174	0.00005
62	-0.02006	0.02586	-0.7755	0.43804
63	0.0485	0.02162	2.24382	0.02484
64	-0.02808	0.0263	-1.06785	0.28559
65	0.3251	0.02553	12.73644	0
66	0.04457	0.0245	1.81896	0.06892
67	0.5558	0.02173	25.57367	0
68	0.01819	0.02123	0.85682	0.39154
69	-0.12486	0.01811	-6.89517	0

21	-0.09141	0.02766	-3.30446	0.00095	70	0.13405	0.02448	5.47583	0
22	0.10386	0.0314	3.30709	0.00094	71	0.08148	0.02705	3.01216	0.00259
23	-0.08707	0.03012	-2.89041	0.00385	72	-0.17741	0.0267	-6.6443	0
24	0.1852	0.02749	6.73608	0	73	0.02161	0.02695	0.80182	0.42266
25	0.03312	0.03072	1.07805	0.28101	74	-0.02931	0.02041	-1.43609	0.15098
26	0.03263	0.02144	1.52172	0.12808	75	0.10254	0.03477	2.94897	0.00319
27	0.10026	0.02262	4.4316	0.00001	76	0.00353	0.03313	0.10643	0.91524
28	-0.03265	0.02191	-1.48993	0.13624	77	-0.1097	0.03261	-3.36403	0.00077
29	0.04931	0.02519	1.95741	0.0503	78	0.01145	0.03566	0.32119	0.74806
30	-0.03772	0.0185	-2.03941	0.04141	79	-0.15419	0.03445	-4.47621	0.00001
31	-0.02882	0.02584	-1.11567	0.26457	80	0.11581	0.03485	3.32309	0.00089
32	-0.08733	0.02437	-3.58314	0.00034	81	0.04288	0.03484	1.23063	0.21846
33	-0.17545	0.01804	-9.72743	0	82	-0.029	0.041	-0.70737	0.47934
34	-0.05184	0.01681	-3.08437	0.00204	83	0.0089	0.03117	0.28559	0.77519
35	-0.06868	0.02206	-3.11396	0.00185	84	-0.24005	0.03002	-7.99587	0
36	-0.02665	0.01782	-1.49531	0.13483	85	-0.16369	0.02376	-6.88832	0
37	0.07795	0.02464	3.16307	0.00156	86	0.24467	0.02275	10.75706	0
38	-0.02416	0.02393	-1.00975	0.31262	87	-0.10891	0.02961	-3.6785	0.00023
39	-0.24092	0.02441	-9.86757	0	88	-0.01344	0.0283	-0.47502	0.63477
40	0.13386	0.02858	4.68451	0	89	0.18316	0.02528	7.24506	0
41	-0.09356	0.02579	-3.6274	0.00029	90	0.0006	0.02837	0.02119	0.9831
42	0.10816	0.0259	4.17609	0.00003	91	-0.04482	0.0282	-1.5893	0.11199
43	0.07551	0.03527	2.14094	0.03228	92	-0.04826	0.02908	-1.65937	0.09704
44	-0.06793	0.02808	-2.41963	0.01554	93	-0.01463	0.02371	-0.61727	0.53706
45	0.0876	0.03668	2.38842	0.01692	94	0.02127	0.02581	0.82403	0.40992
46	0.04724	0.01968	2.40091	0.01635	95	-0.01279	0.02002	-0.63905	0.52279
47	0.13101	0.04325	3.02949	0.00245	96	0.00304	0.02527	0.1202	0.90432
48	-0.05067	0.02323	-2.18101	0.02918					

Movie S1

Video of the subject attempting the graded control muscle contraction experiment with the FES system disabled.

Movie S2

Video of Training Block 1 for the graded control muscle contraction experiment. On the left panel is the video from the overhead camera. The center panel shows cued angle (including the grace period) with the 15° target window on either side. The black line shows the angle the participant is pointing toward. When the pointed angle is within 15° of the cue, the slice will fill with color until either 2 s have elapsed or the participant moves outside the target region. The right panel shows the video processing that leads to the estimated pointing angle.

Movie S3

Video of Generalization Block 1 for the graded control muscle contraction experiment. The panels are the same as in Movie S2. This block demonstrates the participant is able to grade his muscle contraction to point at the three interior angles that he had not attempted previously.
